# Distinct Olfactory Signaling Mechanisms in the Malaria Vector Mosquito *Anopheles gambiae*

**DOI:** 10.1371/journal.pbio.1000467

**Published:** 2010-08-31

**Authors:** Chao Liu, R. Jason Pitts, Jonathan D. Bohbot, Patrick L. Jones, Guirong Wang, Laurence J. Zwiebel

**Affiliations:** 1 Departments of Biological Sciences and Pharmacology, Center for Molecular Neuroscience, Institutes of Chemical Biology and Global Health and Program in Developmental Biology, Vanderbilt University, Nashville, Tennessee, United States of America; 2 USDA, Agricultural Research Service, Henry A. Wallace Beltsville Agricultural Research Center, Plant Sciences Institute, Invasive Insect Biocontrol and Behavior Laboratory, Beltsville, Maryland, United States of America; Texas A&M Health Science Center, United States of America

## Abstract

*Anopheles gambiae* is the principal Afrotropical vector for human malaria, in which olfaction mediates a wide range of both adult and larval behaviors. Indeed, mosquitoes depend on the ability to respond to chemical cues for feeding, host preference, and mate location/selection. Building upon previous work that has characterized a large family of *An. gambiae* odorant receptors (AgORs), we now use behavioral analyses and gene silencing to examine directly the role of AgORs, as well as a newly identified family of candidate chemosensory genes, the *An. gambiae* variant ionotropic receptors (AgIRs), in the larval olfactory system. Our results validate previous studies that directly implicate specific AgORs in behavioral responses to DEET as well as other odorants and reveal the existence of at least two distinct olfactory signaling pathways that are active in *An. gambiae*. One system depends directly on AgORs; the other is AgOR-independent and requires the expression and activity of AgIRs. In addition to clarifying the mechanistic basis for olfaction in this system, these advances may ultimately enhance the development of vector control strategies, targeting olfactory pathways in mosquitoes to reduce the catastrophic effects of malaria and other mosquito-borne diseases.

## Abstract 2

A combination of gene silencing and behavioral studies in the malaria vector mosquito *Anopheles gambiae* sheds light on the olfactory basis of DEET repulsion as well as reveals the role of another family of chemosensory receptors that facilitate olfaction in *An. gambiae*.

## Author Summary

*Anopheles gambiae*, the principal Afrotropical mosquito vector for human malaria, uses olfaction to respond to chemical cues that are required for feeding, host preference, and mate selection. At the heart of this process is a large family of *An. gambiae* odorant receptors (AgORs) that respond to these olfactory cues in peripheral sensory neurons. We have now taken advantage of the relative simplicity of the larval olfactory system to develop a novel behavioral paradigm for individual larva that has been used together with gene silencing approaches in vivo to examine directly the role of AgORs in the olfactory system of *An. gambiae*. In addition to supporting and extending previous studies on the general olfactory response properties of AgORs, our data directly implicate the activity of specific AgORs in mediating the behavioral responses to the commercial insect repellent DEET and reveal the existence of at least two distinct olfactory signaling pathways in *An. gambiae*. One system depends directly on AgORs, while the other is AgOR-independent and requires the expression and activity of a newly identified family of candidate chemosensory genes, the *An. gambiae* variant ionotropic receptors (AgIRs). In addition to clarifying the mechanistic basis for olfaction in this system and the basis of DEET repulsion, these advances may ultimately enhance the development of vector control strategies, targeting olfactory pathways in mosquitoes to reduce the catastrophic effects of malaria and other mosquito-borne diseases.

## Introduction

Chemosensory cues play a central role in directing much of the behavioral repertoire and are a significant determinant in the vectorial capacity of female *An. gambiae* mosquitoes, which are responsible for the transmission of human malaria [1]. Significant progress has been made in identifying the components of olfactory pathways in *An. gambiae* [2],[3],[4],[5],[6]. Nonetheless, there is a paucity of information regarding the precise molecular mechanisms that mediate olfactory signaling in *An. gambiae*.

At the center of the peripheral olfactory signal transduction pathway in *An. gambiae* is a family of odorant receptors (AgORs) that are selectively expressed in olfactory receptor neurons (ORNs). Although originally identified as candidate G-protein-coupled receptors (GPCRs) [7], several studies have disputed the GPCR nature of Anopheline and other insect ORs [8],[9],[10],[11], which likely form ligand-gated heteromeric ion channels that activate ORNs through ionotropic [10] as well as perhaps metabotropic mechanisms [11]. In addition, members of a family of another set of chemosensory receptors related to ionotropic glutamate receptors have recently been described in *Drosophila melanogaster* [12].

The majority of insect ORNs typically express at least two ORs that are likely to form complexes of undetermined stoichiometry that are composed of one highly conserved non-conventional OR83b-like protein together with a conventional OR that presumably mediates odorant binding specificity [4],[8],[13]. In *An. gambiae*, 73 of the 79 AgORs originally identified [7] are expressed in the adult and 13 are expressed in larval stages [14]. The non-conventional anopheline OR83b-like family member, AgOR7, is widely expressed in nearly all olfactory sensilla with the notable exception of grooved-peg sensilla [5], which are activated in vivo by compounds such as ammonia, lactic acid, and other carboxylic acids that are major components of human sweat [15],[16] known to evoke physiological and/or behavioral activity in *An. gambiae* [17],[18]. Indeed, recent functional analyses of AgOR odor space reveal a paucity of responses for these groups of odorants, suggesting anopheline sensitivity to amines and other variant odorants may lie outside of AgOR-based signaling [17],[18].

In order to improve our understanding of mosquito olfaction, we have continued to utilize the relative simplicity of the *An. gambiae* larval olfactory system, which consists of only 12 ORNs [14]. In previous studies utilizing behavioral and functional approaches to describe the molecular and cellular basis for olfactory responses to a range of natural and synthetic chemical stimuli, we identified a subset of AgORs expressed in the larval antenna that are tuned to odorants that elicit specific behavioral responses [14]. Building upon those studies, we now use RNAi-based gene-silencing approaches to validate in vivo the role of AgORs in larval olfactory signal transduction and specifically identify the molecular receptor that mediates the repellent activity of N, N-diethyl-m-toluamide (DEET). In addition, we have identified and characterized a family of chemosensory receptors that are related to inotropic glutamate receptors (AgIRs) that underlie a novel-signaling pathway that is independent of AgOR activity. We propose that *An. gambiae* expresses distinct signaling pathways that participate in larval olfaction and are likely to also be active in mediating adult responses to a diverse range of chemosensory stimuli. These studies further our understanding of the molecular basis of olfaction and olfactory-driven behaviors in *An. gambiae* and lay the foundation for advancing alternatives to mosquito control strategies focused on adult life stages.

## Materials and Methods

### Mosquito Rearing

*An. gambiae sensu stricto*, originated from Suakoko, Liberia, was reared as described [2]. For stock propagation, 4- to 5-d-old female mosquitoes were blood fed for 30–45 min on anesthetized mice, following the guidelines set by Vanderbilt Institutional Animal Care and Use Committee.

### Individual Larval Behavioral Assays

Larval assays were conducted between ZT2 and ZT10 during the standard LD12∶12 rearing cycle. Here, *An. gambiae* 2^nd^ or 3^rd^ instar larvae were removed from rearing pans, rinsed carefully with distilled water to eliminate any remaining food residue, and kept in segregated containers with distilled water for 30 min. Odorant stocks were made by dissolving odorant (>99% pure or of the highest grade commercially available) in pre-heated (70°C) 2% NuSieve, GTG low-melting-temperature agarose (Cambrex Bio Science). The assay was performed in a 10×1.5 cm Petri dish containing 50 ml of 27°C distilled water. The odorant and larva dropping spots were located at opposite ends along the diameter and marked by a solid circle and a cross, respectively. The odorant/control stock was placed into the dish for 1 min beforehand to equilibrate, and the larva was gently introduced at the marked spot.

**Figure 1:**
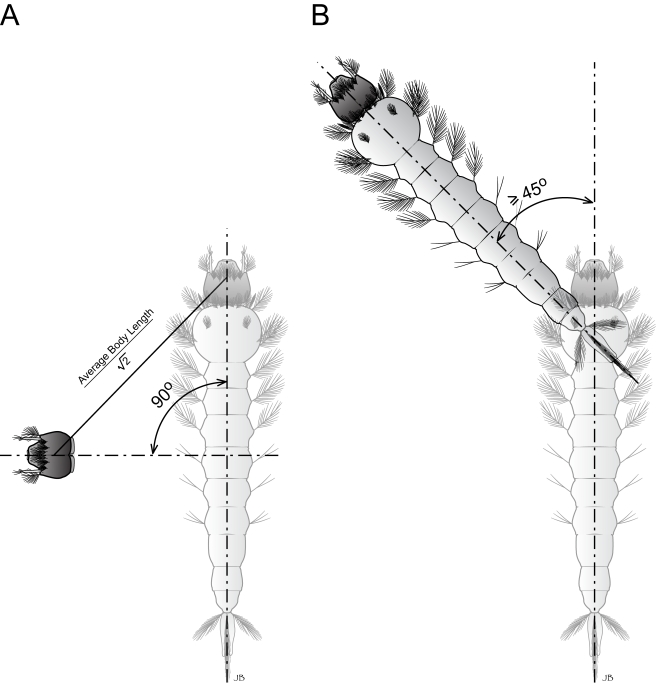
Operational definitions of larval movements and turns. (A) A larval body movement threshold is characterized by a larva turning its body axis by 90° and its head traveling the distance indicated. (B) A larval turn threshold is defined by a 45° angle between two successive larval tracking vectors.

Real-time images of larval movements were obtained and downloaded at 1 s intervals for the duration of the 5 min assay using a custom-designed 30 frames/s video camera/computer/software system (Model NC-70, DAGE-MTI, Michigan City, Apple PowerMac 8500/Scion Image J v1.63, National Institutes of Health, USA). At the conclusion of each assay, all larvae were individually stored at −80°C for molecular analyses, as described below. The images were subsequently sorted and analyzed using Image J (version 1.40g, NIH, USA) with its Mtrack J plug-in (version 1.3.0). The analysis of larval responses was carried out by tracking the motion of individual larva after marking the position of the larva's anterior, which was easily discernable in our system. In this manner, we were able to monitor and calculate the number of larval turns, overall movement, resting time (s), and average velocity (mm/s) to provide a comprehensive characterization of larval behavior patterns. Similarly, a turn threshold was defined such that if the intersection angle between two successive larval tracking vectors exceeded 45°, the larvae were considered to have carried out a turn (Figure 1). Similarly, movement thresholds were defined so as to recognize false movements and account for the tendency of *An. gambiae* larvae to stochastically perform body swirls that appear to lack any horizontal locomotion. In our hands, a movement threshold was set by establishing that an individual larva turns 90° relative to an axis set at the body-length midpoint; the distance between the previous and the current position of the larval head can be calculated using the equation: 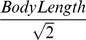. By setting the movement threshold in such a manner, we were able to compensate for false movements that result from the tendency of *An. gambiae* larvae to stochastically perform body swirls that appear to lack any horizontal locomotion. After measurement of multiple (*n*>30) stage-2 and stage-3 larvae, we calculated the average larval body length as ∼3.25 mm in our CCD system, thereby establishing a threshold for larval movements at ∼2.3 mm, such that any shift in larval head position exceeding this value was defined as a single instance of larval movement (Figure 1). In addition to analyzing tracking data for the number of movements and turns, we also measured the average velocity (mm/s) and resting time (s) over the course of the entire assay. Arithmetic means for each assay/treatment were analyzed for statistical significance using single-factor ANOVA; significant results were followed up with Tukey-Kramer post-tests to distinguish among groups using JMP software (v. 4.0.4, SAS, Cary, NC). In the cases where antennal and maxillary palp ablations of larvae were conducted, all manipulations were carried out by manual dissection at 2^nd^ instar stages, after which larvae were allowed to recover for 24 h prior to behavioral testing.

### AgIR Identification and Expression

Candidate AgIR sequences were identified in both the *An. gambiae* genome using DmIR amino acid sequences as tBLASTn and BLASTp queries, respectively. Potential exon-intron gene models were predicted based on homology to DmIRs or AgIRs, as well as with the aid of a Hidden Markov Model-based gene structure predictor (www.Softberry.com). Iterative searches of all gene models were carried out until no new candidates were identified. Conceptual translations of full AgIR coding sequences were aligned with DmIR protein sequences using Clustal X. Phylogenetic trees were constructed using the Neighbor-Joining method [19] with bootstrap resampling of 1,000 pseudo-replicates. Transmembrane helices were predicted using Hidden Markov Model-based software from the Center for Biological Sequence Analysis (Technical University of Denmark, http://www.cbs.dtu.dk/services/TMHMM-2.0/). Antennae from late-instar *An. gambiae* larvae were hand-dissected into RNA*Later*-Ice solution (Ambion, Austin, TX). Total RNA extraction and cDNA synthesis were performed using the RNeasy Mini (Qiagen) and Transcriptor First Strand cDNA Synthesis (Roche) kits, respectively. Antennal cDNA was used as a template in PCR as described [2]. PCR primers specific for *AgIr*s were as follows: *AgIr8a*: f5′-CCCTATGAGTGCAGAAAATT-3′ and r5′-GGTACAGCACGTCTTCTGCG-3′; *AgIr25a*: f5′-CAACCGACATACGCTACCAA-3′ and r5′-ACGATGAATACGCCTCCGAT-3′; AgIr41a: *f5′-ACTGGGAACTGGAGGTGGTG-3′* and r5′-CTAAGGTGTCTCACTCCTCC-3′; *AgIr41n* f5′-ATGCACGATACATCTTGCCG-3′ and r5′-TAAAGGACAGGAACGGTGTG-3′; *AgIr76b*: f5′-CACGCTCCCAATCAACAATG-3′ and r5′-GATGGCGGCTAAACACTTCC-3′; *AgNMDAR2* f5′-AAGTTGGTGCTATGGATCAT-3′ and r5′-ACACCATACGCATATACCCG-3′; *rps7* f5′-GGCGATCATCATCTACGTGC-3′ and r5′-GTAGCTGCTGCAAACTTCGG-3′. cDNA amplicons were TOPO-TA cloned into plasmid pCRII (Invitrogen) and sequenced to confirm their identities.

### siRNA Preparation and Injection

Double-stranded (ds) RNAs against a specific target gene were prepared and purified using bidirectional in vitro transcription of full-length cDNA templates using flanking T7 transcription initiation sites, and siRNAs were prepared via RNAse III digestion using Silencer siRNA Construction reagents and protocols (Applied BioSystems/Ambion, Austin, TX). Healthy, wild-type 2^nd^ instar *An. gambiae* larvae were chosen for micro-injection. They were pre-immobilized on 3MM filter paper on top of a 4°C chill platform (BioQuip Inc, Rancho Dominquez, CA). Additional desiccation was achieved using Kimwipes (Kimberly-Clark, Dallas TX) to gently dry individual larva. Twin styrofoam strips were also employed as temperature sinks to reduce distress from cold temperatures. Single barrel borosilicate glass capillary pipettes (World Precision Instruments, Sarasota, FL) were pulled (using a P-97 puller, Sutter Instruments, Novato, CA) and beveled (using a Narishige EG-5 beveller, Tokyo, Japan) to form microinjection needles. For larval microinjection, 27.6 nl of 100 nM siRNA were injected into the dorsal side of the larval thorax using a Nanoliter 2000 system (World Precision Instruments, Sarasota, FL). Post-injection, larvae were allowed to recover in 27°C distilled H_2_0 with 1 ml of larval food (as described in Mosquito Rearing section) for 48 h. Larvae were monitored every 24 h post-injection, and non-viable individuals were discarded.

### Real-Time PCR (qRT-PCR)

Subsequent to experimental treatments and behavioral assays, *AgOr7*, *AgOr40* and *AgIr76b* transcript levels were determined by means of quantitative RT-PCR. Each sample was comprised of 10 (*AgOr7*) or 30 (*AgOr40*, *AgIr76b*) larval heads that were hand-dissected from batches of control and experimental *An. gambiae* larvae. RNA extraction and cDNA synthesis were performed using the QIAGEN RNeasy Mini Kit and Roche Transcriptor First Strand cDNA Synthesis Kit, respectively. All primers in the assay were designed to span predicted introns in order to distinguish well between genomic DNA and cDNA templates. *An. gambiae* ribosomal protein S7 (*rps7*), which is constitutively expressed at high levels in all tissues, was chosen as control gene to measure the relative levels of mRNA of target genes in vivo. Primer sequences are as follows: *rps7*, f5′-GGCGATCATCATCTACGTGC-3′ and r5′-GTAGCTGCTGCAAACTTCGG-3′ (product size: 458bp cDNA); *AgOr7*, f5′-ATCTTTGGCAATCGGCTCATC-3′ and r5′-GGCTCCAAGAACCGAAGC-3′ (product size: 346 bp cDNA); *AgOr40*, f5′-GACCCTCAAGAACCAGGGCT-3′ and r5′-AATGATGGTGTAGTACGAGAAGG-3′; *AgIr76b*, f5′-ATCTTCGATCCAGAGTTGCT-3′ and r5′-CCGGTCACCATGACGAAGTA-3′. qRT-PCR was carried out using an Applied Biosystems 7300 Real-time PCR system and SYBR green as fluorescent dye. Three experimental repetitions were analyzed for each biological sample and the data processed using System 7300 Sequence Detection Software (version 1.3.1). Primer efficiency was determined using a standard curve for all the primers used. In the amplification of target genes and *Rps7*, 8 µl and 2 µl cDNA, respectively, from each group were used as templates. In each trial, cDNA levels of target genes were quantified relative to r*ps7* levels using the method of Pfaffl [20].

## Results

### Behavioral Responses of Individual Larva

**Figure 2:**
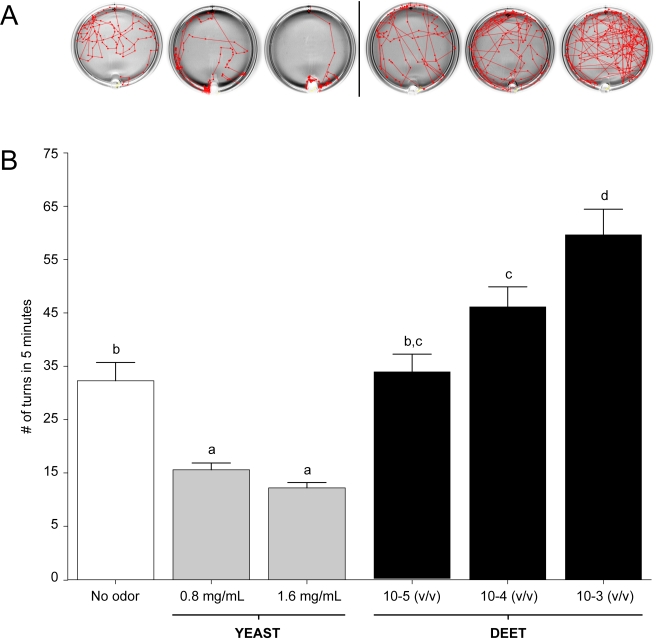
Larval responses in *An. gambiae* to yeast and DEET elicit opposite behaviors. (A) 2-D tracking maps (top view) of freely moving individual larva during a 5 min time lapse. (B) Average number of turns exhibited by larvae in response to no odor, two concentrations of yeast paste, and three concentrations of DEET were assessed independently over a 5 min time lapse. Treatments with high DEET concentrations (10^−4^ and 10^−3^ v/v dilutions) and yeast paste (0.8 and 1.6 mg/ml) differed significantly from the no-odor control (*p*<0.01). Results are shown as means ± SE, *n* = 10.

**Figure 3:**
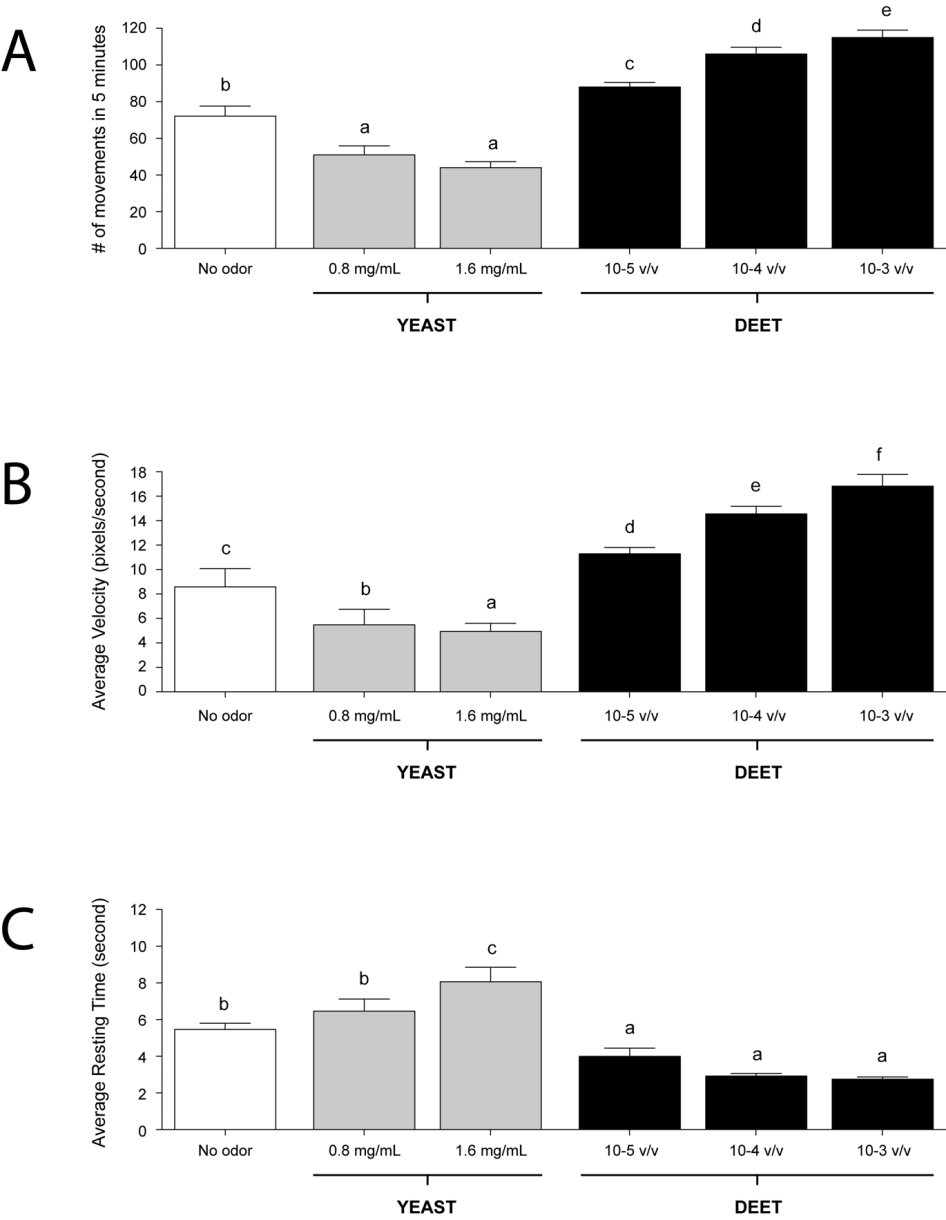
Behavioral effects of yeast and DEET on *An. gambiae*. Larval responses to yeast and DEET stimuli. Average number of movements (A), velocity (B), and resting time (C)—histograms of larval responses to two concentrations of yeast paste and three concentrations of DEET. Compared with the no-odor control, yeast, and DEET significantly affected larval activity (*p*<0.05). Results are shown as means ± SE, *n* = 10.

Previous studies utilized a novel paradigm to assay the behavioral responses of large groups of *An. gambiae* late instar larvae to various natural and synthetic odorants in order to characterize the molecular and cellular elements of the larval olfactory system [14]. While providing fundamental information about the components underlying the olfactory responses of *An. gambiae* larvae, these end-point studies did not provide the precise tracking information that would allow us to distinguish between attractive or repulsive behavioral patterns. In addition, the need for a large number of larvae precluded its use in other experimental contexts. To provide such information and utility, a CCD camera-based tracking system was utilized to study the behavior of individual *An. gambiae* larva in response to odorant stimuli. Visual tracking records (Figure 2) were then analyzed to distinguish parameters associated with directional movement. These included calculating the total number of turns, the overall number of movements, the average velocity, and the resting time for each larval behavioral assay (Figures 2 and 3).

The sensitivity of this system was initially tested with two odorant stimuli, each of which evoked a strong dose-dependent response in the *An. gambiae* larvae group assay paradigm [14]. The first was DEET, which is a widely used commercial insect repellent. The second was yeast paste, a complex odorant source and a normal component of larval food [14]. The behavioral responses of individual *An. gambiae* larva to three concentrations of DEET and two concentrations of yeast paste were examined along with the appropriate set of parallel no-odorant controls (Figure 2). For each assay, the four behavioral parameters described above were quantified. In these studies, yeast paste elicited decreases in overall larval turning (inverse klinokinesis; Figure 2) and movement (Figure 3) as well as concomitant increases in resting time when compared with no-odorant controls. In contrast, DEET elicited nearly the opposite effect: *An. gambiae* larvae displayed a dose-dependent increase in the turning rate (direct klinokinesis; Figure 2), number of movements, and average velocity (direct orthokinesis; Figure 3), while the average resting time was reduced to threshold levels at dilutions of 10^−3^ and 10^−4^.

**Figure 4:**
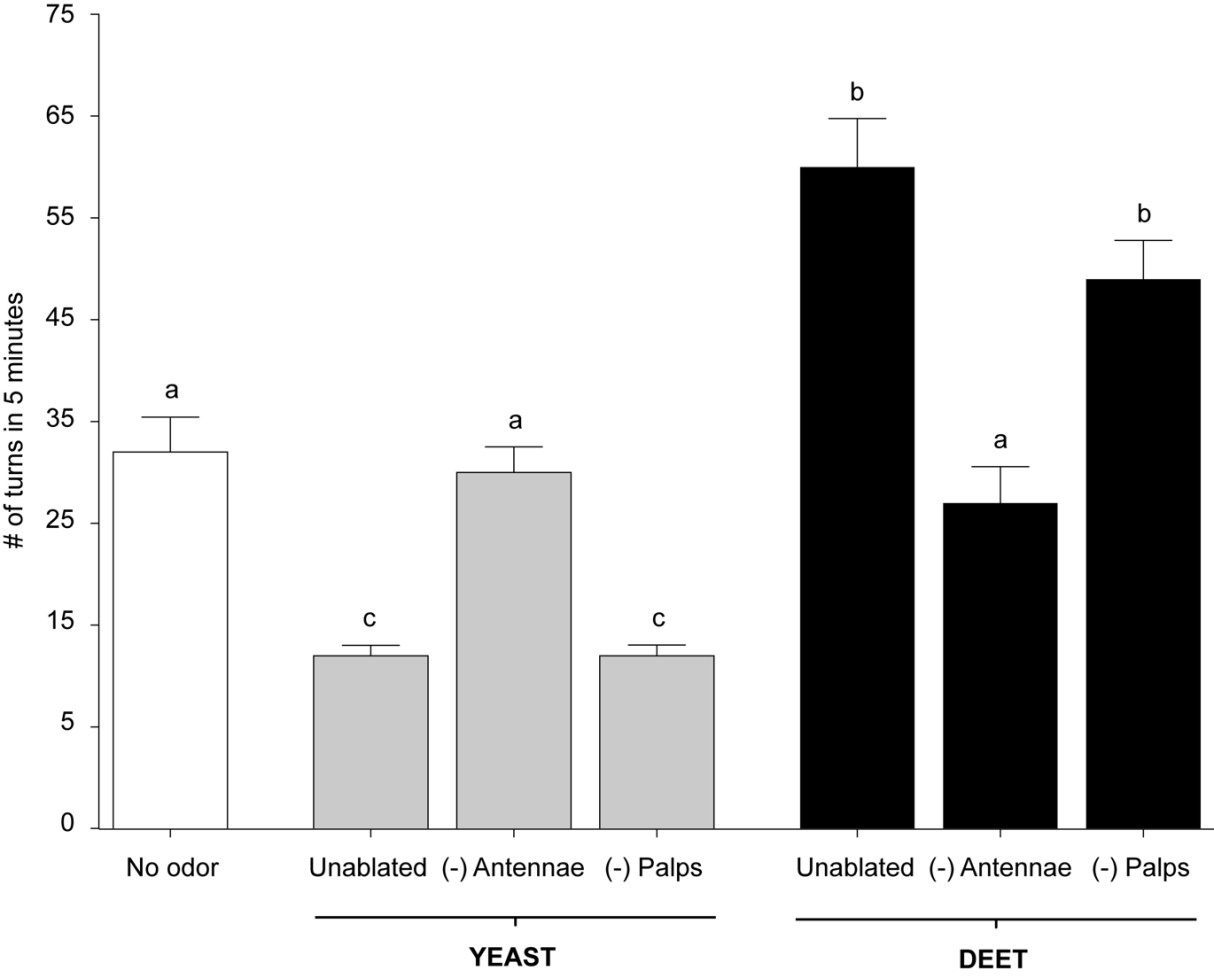
Larval antennae mediate responses to yeast and DEET. In the presence of yeast and DEET, unablated and palp-ablated larvae responded equally to both; ablation of the antennae, however, significantly increased or decreased the number of turns (*p*<0.05) in response to yeast and DEET, respectively. Results are shown as means ± SE, *n* = 10.

To confirm that the odorant-evoked behavioral responses were mediated by the larval olfactory system, a parallel set of assays were carried out after hand dissection of both larval antennae to effectively eliminate the site of olfactory signal transduction. Antennal-ablated larvae appeared to be largely indifferent to high concentrations of both DEET and yeast, as larval responses were indistinguishable from no-odorant and unablated controls (Figure 4). In larvae in which the antennae were left intact but maxillary palps removed, responses to DEET and yeast paste were similar to those in unablated controls (Figure 4). Taken together, these data demonstrate that we have developed a robust behavioral paradigm for examining odorant-induced responses from individual *An. gambiae* larva.

### AgORs Silencing Confirms a Direct Role in the DEET Response

To discern the molecular basis for odorant-evoked behavioral responses of *An. gambiae* larvae, we initially focused on the role of *AgOr7*, which is the *An. gambiae* ortholog of the non-conventional *Drosophila* OR, *DmOr83b* [5],[7], and is highly expressed in the larval antenna [14]. In the absence of effective strategies to generate mutant or transgenic strains of *An. gambiae*, we used RNA interference (RNAi) to reduce *AgOr7* mRNA levels in individual larva, which could then be tested for abnormal behavioral responses. Individual larval behavioral assays followed by quantitative RNA analyses were conducted to assess the effects of *AgOr*7 siRNA and control siRNA microinjections on olfactory responses and transcript levels. To account for non-specific effects of siRNA delivery, larvae were microinjected with identical amounts of a siRNA designed against a gene (AT5G39360) from the *Arabidopsis thaliana* genome lacking significant homology to any cDNA in *An. gambiae*. Furthermore, buffer-alone microinjections were carried out in parallel to assess any potential effects of microinjection on larval behavior.

In order to assess the efficiency of siRNA-mediated knockdown of *AgOr7* transcripts, a series of qRT-PCR studies were carried out on experimental and control larvae after behavioral testing. In these assays, cDNA was prepared from larval heads (with olfactory antennae attached) from individual larva collected immediately following behavioral testing. These data (Figure S1) confirm that microinjection of siRNAs targeting *AgOr7* resulted in dramatic decreases in levels of this transcript.

**Figure 5:**
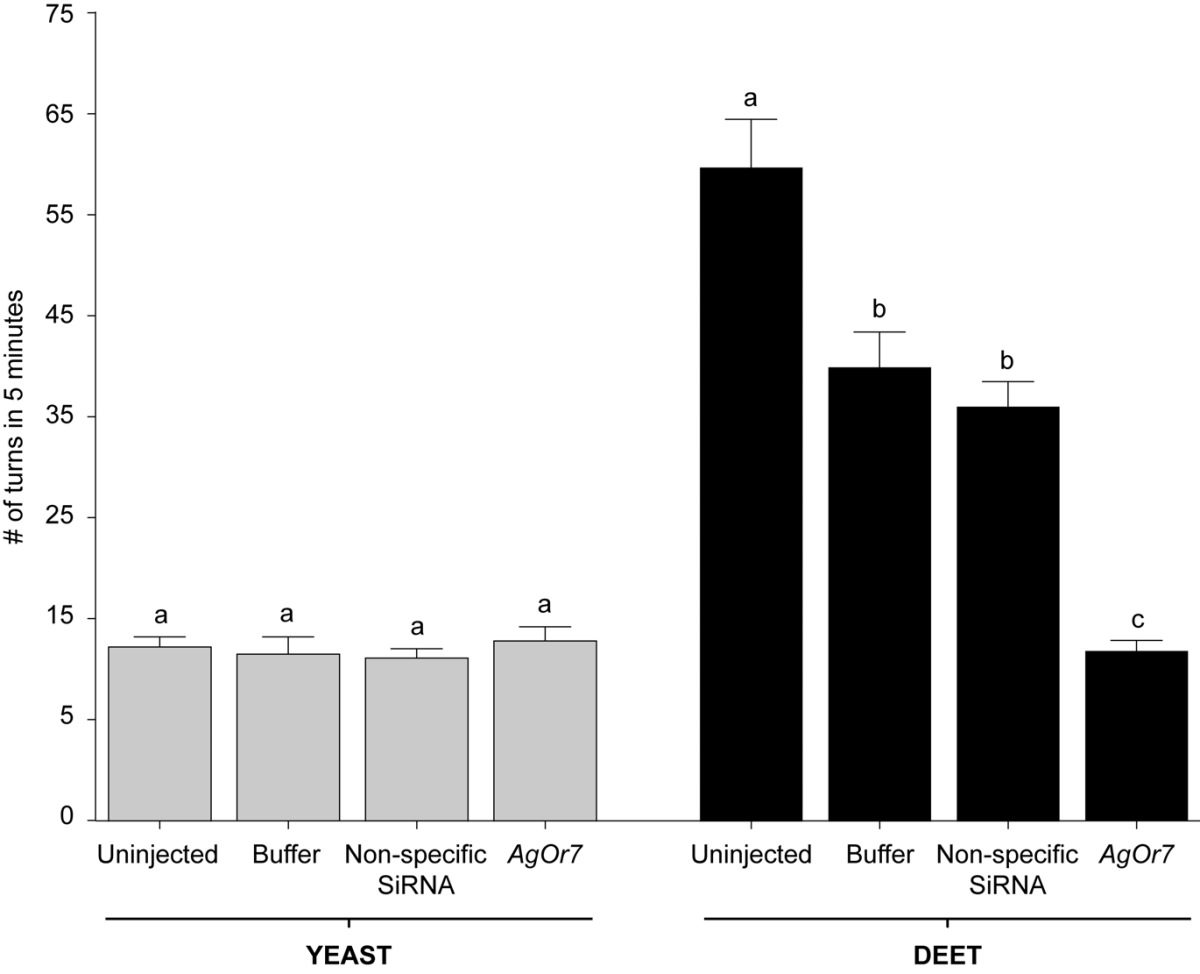
Differential sensitivity of larval responses in *An. gambiae* to siRNA-mediated knockdown of *AgOr7* is odorant dependent. The average number of turns exhibited by uninjected larvae as well as those receiving mock (buffer-alone), non-specific, or siRNA injections in response to yeast paste and DEET were assessed independently over a 5 min time lapse. Larval responses to 1.6 mg/ml yeast paste were unaffected by any siRNA treatments (A) while larvae receiving *AgOr7* siRNAs displayed significant reductions in turning rates in response to a 10^−3^ v/v dilution of DEET (B). Buffer and non-specific siRNA-injected animals displayed a comparable reduction of the number of turns (*p*<0.05). Results are shown as means ± SE, *n* = 10.

**Figure 6:**
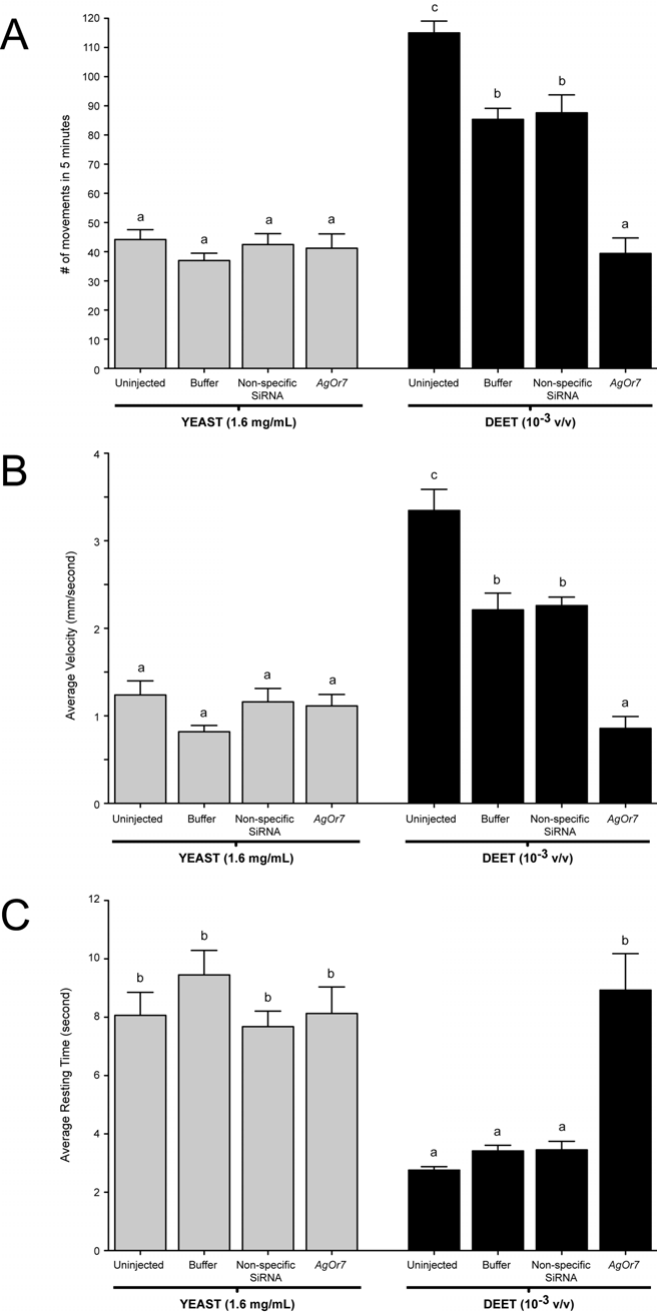
Larval behaviors after injection of non-specific small interfering RNA (siRNA). Averaged responses of buffer, non-specific, and *AgOr7* siRNA-injected larvae in the presence of 1.6 mg/ml yeast paste and a 10^−3^ v/v dilution of DEET. Larval movement (A), velocity (B), and resting time (C) behaviors of larvae in response to yeast paste and DEET. Knockdown of *AgOr7* mRNA levels has no effect on the ability of larvae to respond to yeast paste yet evokes significant behavioral alterations in larval responses to DEET (*p*<0.01). Results are shown as means ± SE, *n* = 10.

Although a modest microinjection effect was observed on the average larval velocity, the overall number of turns (Figure 5) as well as the number of movements, average velocity, and resting time (Figure 6) in response to 1.6 mg/ml yeast paste stimuli were largely unaffected by microinjection with *AgOr7* or control siRNAs. In contrast, a 1×10^−3^ (v/v) dilution of DEET in individuals that received *AgOr7* siRNA showed significant (*p*<0.01) reductions in turns (Figure 5), movements, and velocity as well as a significant increase in their average resting time relative to buffer-injected and control larvae (Figure 6). Although a modest microinjection effect was again observed in buffer-injected larvae, these results are consistent with the hypothesis that larval responses to DEET are *AgOr7*-dependent whilst larval responses to yeast paste are *AgOr7*-independent.

**Figure 7:**
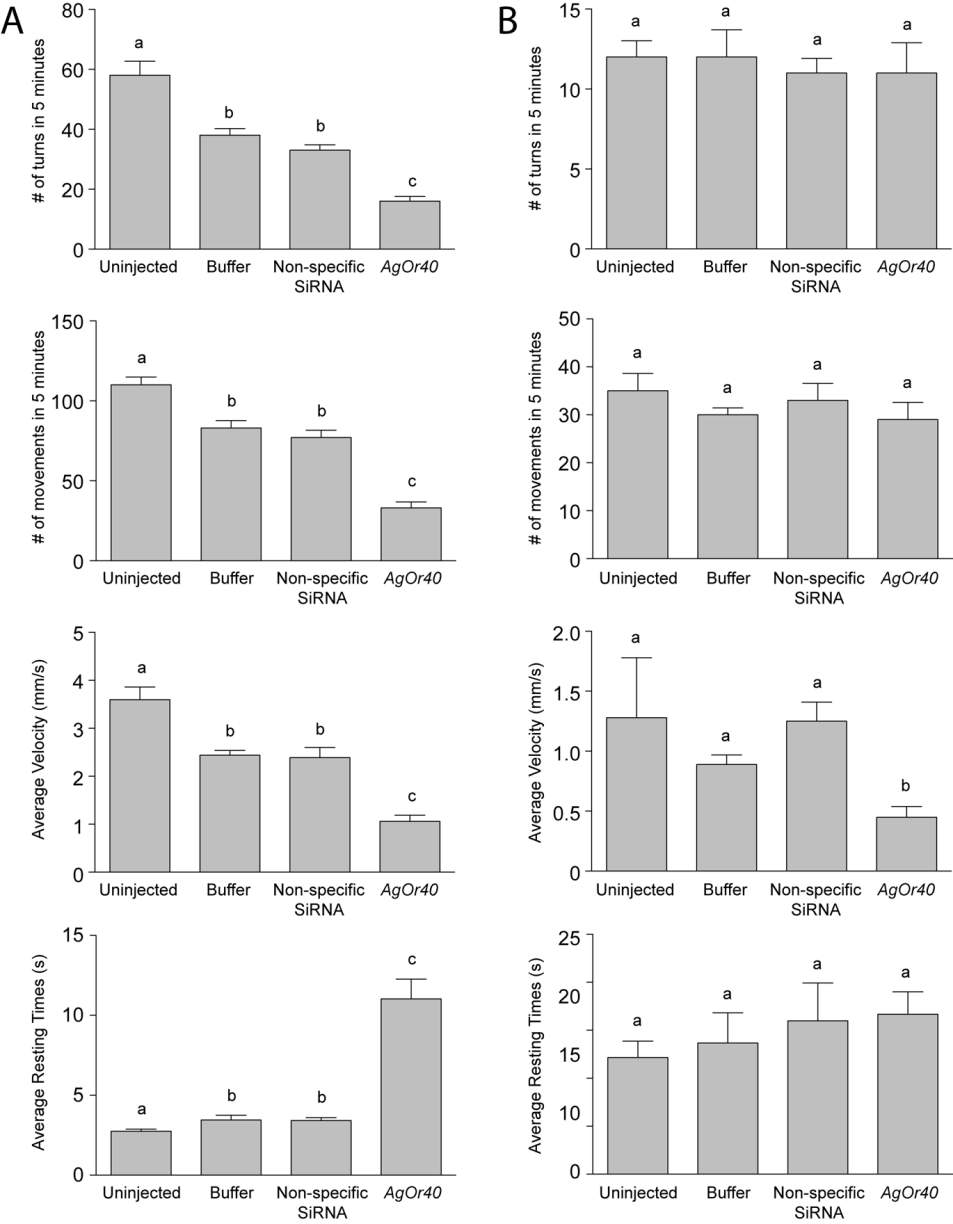
Differential sensitivity of larval responses in *An. gambiae* to siRNA-mediated knockdown of *AgOr40* is odorant dependent. Larval responses exhibited by uninjected larvae as well as those receiving mock (buffer-alone), non-specific, or siRNA injections in response to DEET (A) and yeast paste (B) were assessed independently over a 5 min time lapse. Larval responses to 1.6 mg/ml yeast paste were unaffected by any siRNA treatments while larvae receiving *AgOr40* siRNAs displayed significant reductions in turning rates (top panel) in response to a 10^−3^ v/v dilution of DEET. Buffer and non-specific siRNA-injected animals displayed a comparable reduction of the number of turns (*p*<0.05). Larval movement, velocity, and resting time behaviors (from top to bottom) of larvae in response to DEET (A) and yeast paste (B) where knockdown of *AgOr40* mRNA levels had no effect on the ability of larvae to respond to yeast paste yet evoked significant behavioral alterations in larval responses to DEET (*p*<0.01). Results are shown as means ± SE, *n* = 10.

Functional studies using Xenopus oocytes [14] have previously identified AgOR40 as a conventional ligand-specific larval AgOR that responds to DEET stimulation and, by implication, is likely to be responsible for DEET-elicited behavioral responses in *An. gambiae* larvae. Inasmuch as the molecular basis for DEET mediated behaviors remains controversial, we tested this hypothesis by using siRNA-mediated gene silencing to examine whether knockdown of *AgOr40* transcripts would also perturb behavioral responses to DEET and yeast paste. In these studies, injection of siRNAs targeting *AgOr40* echoed the effects of *AgOr7* siRNAs and showed a significant reduction in turns and other elements of larval behavior in response to DEET stimuli (Figure 7A) and were unaffected in response to yeast paste (Figure 7B). As was the case for *AgOr7* silencing, qRT-PCR studies were carried out on experimental and control larvae after behavioral testing to assess the levels of *AgOr40* transcripts. These data (Figure S1) confirm that microinjection of siRNAs targeting *AgOr40* resulted in dramatic decreases in *AgOr40* transcript levels without significantly altering *AgOr7* mRNA pools. Taken together, these data directly validate the role of AgOR40 as a DEET-specific conventional AgOR in the larval olfactory system of *An. gambiae*.

### AgIRs Mediate AgOR Independent Olfactory Responses

Based on the *AgOr7*-independent response of larvae to yeast paste, we next investigated whether *AgOr7*-dependent and -independent olfactory signaling exists in *An. gambiae* larvae. In doing so, we considered that *AgOr7* independence of the larval yeast response might, in part, reflect that yeast paste is a complex mixture, some components of which may activate *AgOr7*-independent olfactory signaling pathways. In contrast, DEET is a unitary compound that specifically elicits *AgOr*-dependent behavioral responses in *An. gambiae* larvae and physiological responses in Xenopus oocyte-based AgOR functional assays [14].

To examine further the possibility that distinct signaling pathways are active in this system, we searched the *An. gambiae* genome for homologs of variant ionotropic glutamate receptors that have recently been shown to function as novel chemosensory proteins in *D. melanogaster* (DmIRs) [12]. We have identified a family of 46 *An. gambiae* variant ionotropic glutamate receptors, which we have named *AgamGLUVIRs*, and 9 homologs of ionotropic glutamate receptors, named *AgamGLURs* or *AgamNMDARs*, all according to the convention established by the *An. gambiae* genome consortium (www.Vectorbase.org). For convenience we refer to the *AgamGLUVIR* genes as *AgIrs* and their conceptual peptide products as AgIRs. Another group of researchers has independently identified the same family of genes [21] and we have agreed with them on a unified nomenclature in order to avoid confusion in future publications. A listing of the entire gene family, their chromosome positions, and peptide sequences is given in a Table S1.

**Figure 8:**
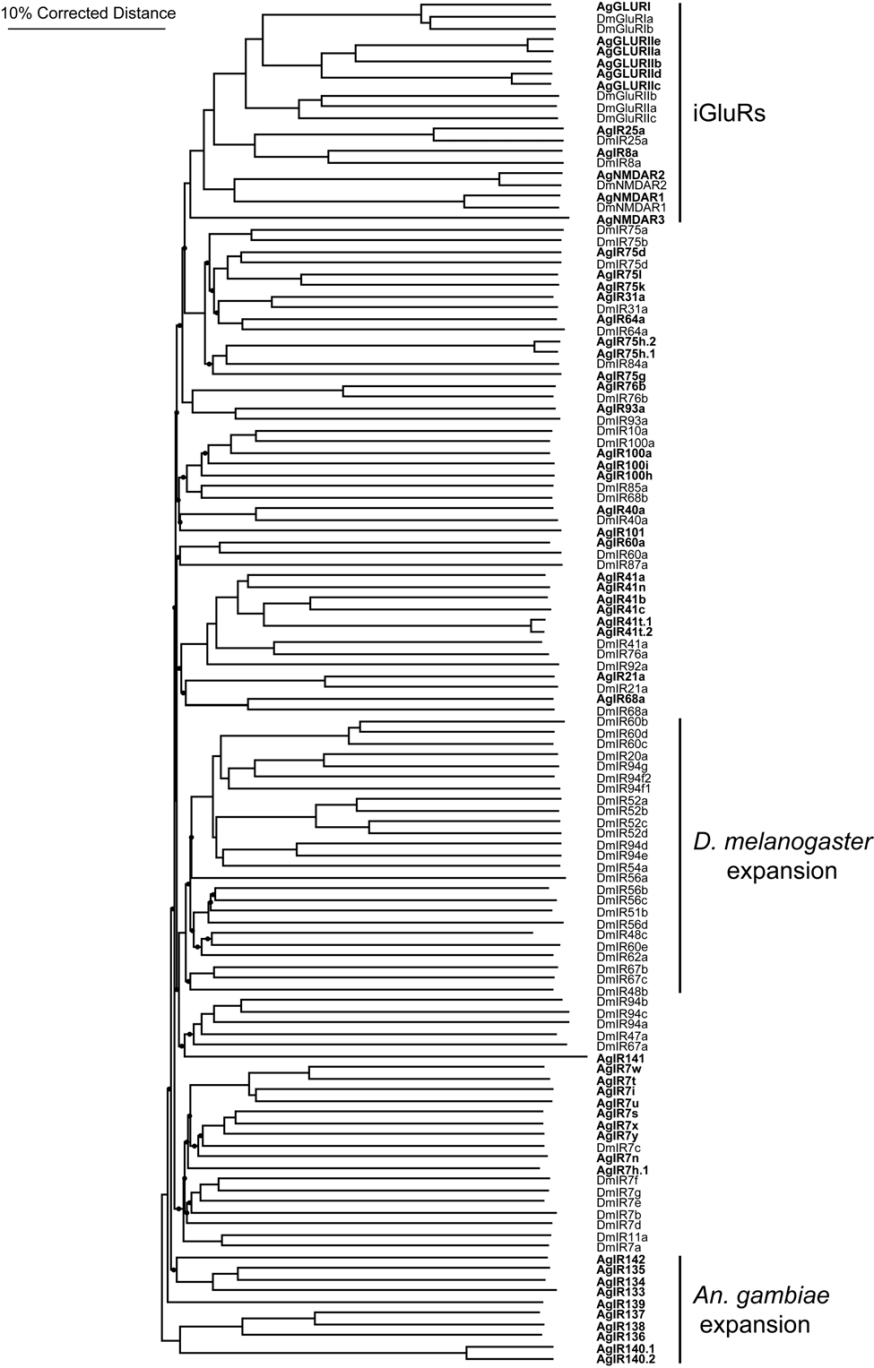
AgIR/DmIR phylogenetic tree. Neighbor-joining tree based on amino acid alignments of AgIR and DmIR peptides. AgIR names are shown in bold type and DmIR names are shown in plain type. Black dots indicate branch points where bootstrap support is less than 50%.

**Figure 9:**
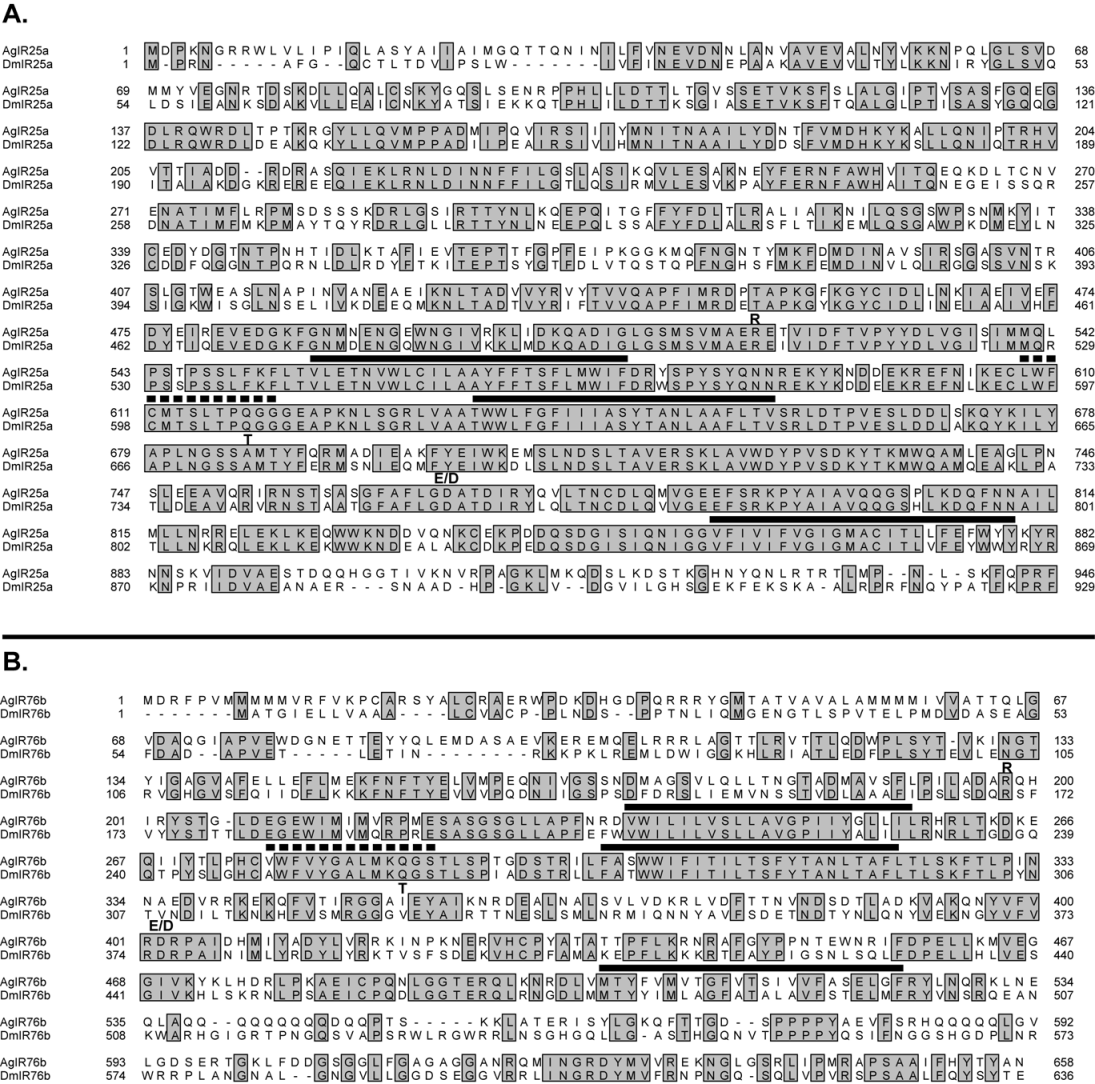
Representative alignments of AgIR and DmIR homologs. (A) IR25a peptide alignment. (B) IR76b peptide alignment. Amino acid sequences (single letter code) were aligned using ClustalX. Identical residues are shaded. Bold lines above residues indicate predicted transmembrane helices, while the dotted line above residues indicates the potential pore loop. Boldface letters represent amino acids arginine (R), threonine (T), or glutamic acid/aspartic acid (E/D) at positions that are found in known glutamate receptors.

A phylogenetic reconstruction comparing the amino acid sequences of AgIRs and DmIRs shows deep branching and low bootstrap support for many of the implied relationships, reflecting the considerable sequence diversity between these proteins both within and across species (Figure 8). The most convincing relationships are observed within the iGluRs, suggesting conservation of function (Figure 8). Very few strong homologs are observed between AgIRs and DmIRs. Despite their diversity, topology predictions indicate conservation of 4 hydrophobic stretches of amino acids that likely correlate to the transmembrane and pore regions (Figure 9) of known ionotropic glutamate receptors (for review see [22]).

Interestingly, two of the strongest AgIR homologs of DmIRs are found within the iGluR clade (Figure 8). AgIR25a shares 68% amino acid identity (84% similarity) with DmIR25a, and AgIR8a shares 42% identity (63% similarity) with DmIR8a, genes that are broadly expressed in coeloconic sensilla neurons in the third antennal segment of *D. melanogaster* [12]. These 2 peptides are also much longer, 891aa and 946aa, respectively, than other AgIRs (average length 664aa) and are closer in size to the iGluRs (avg. 974aa, including partial peptides). Moreover, AgIR25 has retained 2 of the 3 amino acids, an arginine and an aspartic acid (Figure 9A), in positions that are known to be important for glutamate binding [22]. Importantly, some classes of NMDA receptors also lack the 3rd residue [22]. AgIR8a has potential glutamate-binding residues in all three conserved positions, while several other AgIRs, including AgIR76b, retain one or more (Figure 9B). Most other AgIRs are divergent at those positions (unpublished data, Table S1).

**Figure 10:**
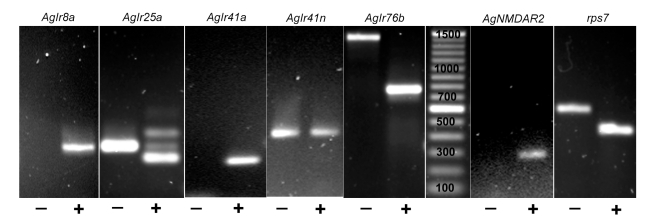
Expression of *AgIrs* in larval antennae. Composite image of agarose gel lanes showing cDNA (lower) and gDNA (upper) bands following RT-PCR using *AgIr*-specific primers as indicated above lanes. Minus (−) and plus (+) signs below lanes indicate the presence or absence of reverse transcriptase in first strand cDNA synthesis reaction, respectively. Bands (base pairs): *AgIr8a* cDNA (319); *AgIr25a* cDNA (271), gDNA (334); *AgIr41a* cDNA (245); *AgIr41n* cDNA (336, not present), gDNA (417); *AgNDMAR2* cDNA (328); *AgIr76b* cDNA (770), gDNA (1414); *rps7* cDNA (460), gDNA (609). No genomic bands were expected for *AgIr8a*, *AgIr41a*, and *AgNDMAR2* as the forward primers spanned an exon-exon junction. All bands that appeared in gels are shown and Photoshop was used only to adjust the brightness and contrast of each panel. Marker lane shows 100 bp ladder (New England Biolabs).

As a first step toward characterizing the potential role of AgIRs in larval olfactory signaling, we carried out RT-PCR using cDNA derived from *An. gambiae* larval antennae and gene-specific primers to 5 *AgIr* genes. These studies indicated that multiple members of this class of candidate chemosensory genes are expressed in the larval antenna (Figure 10) as 4 of the 5 *AgIrs* could be amplified from larval antennae. Additionally, expression of one member of the ionotropic glutamate receptor family, *AgNMDAR2*, was observed in larval antennae (Figure 10). We expect future work to elucidate the expression profiles of all *AgIr*s in both the larval and adult olfactory tissues of *An. gambiae*.

**Figure 11:**
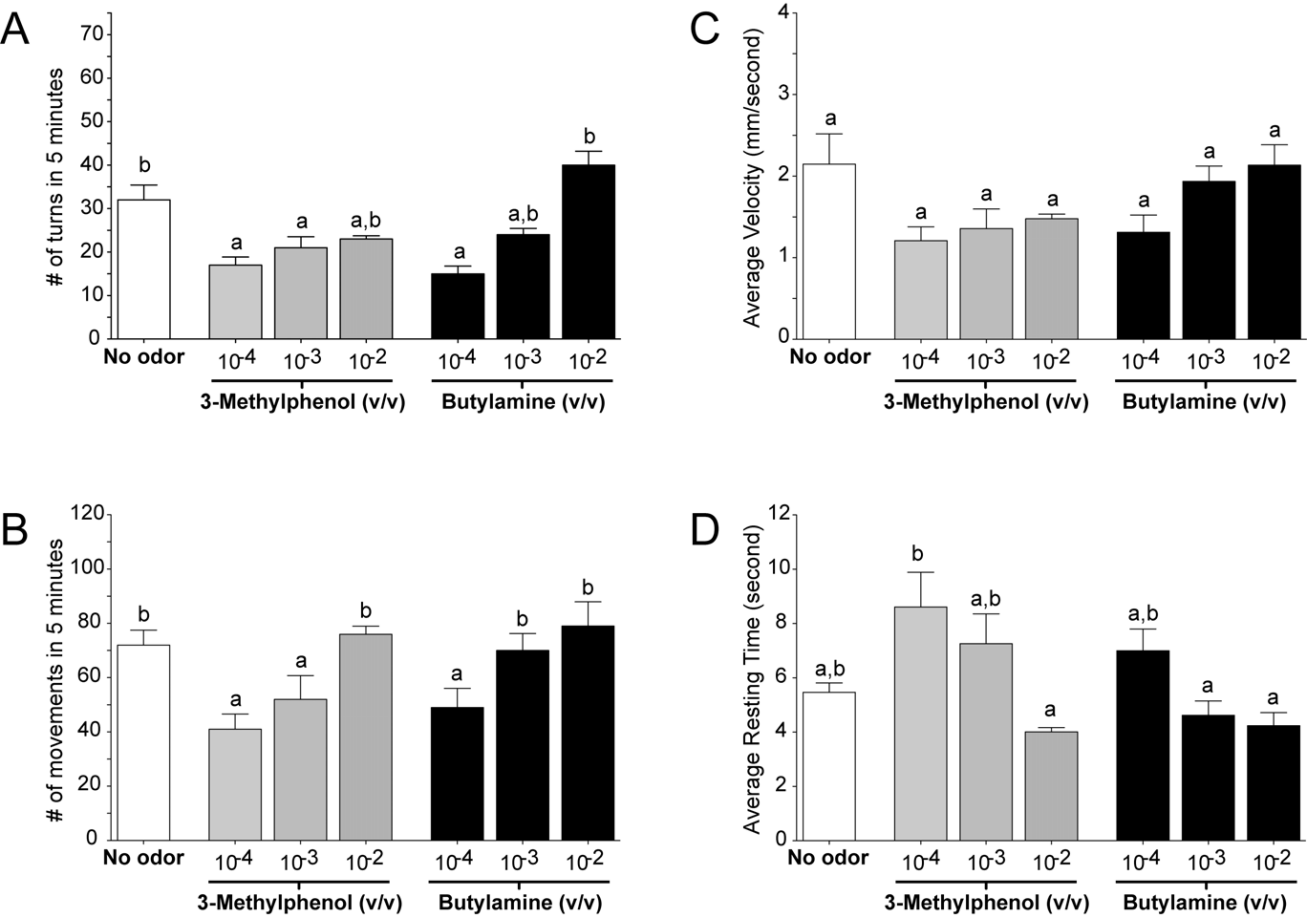
Behavioral effects of 3MP and Butylamine on *An. gambiae*. Larval responses to increasing dilutions (v/v) of 3MP and butylamine are displayed: total number of turns/assay (A), average number of movements/assay (B), average velocity (C), and resting time (D). With the exception of average velocity, for which no significant effects were detected, both odorants evoked dose-dependent responses on larval activity when compared with the no-odor control (*p*<0.05). Results are shown as means ± SE, *n* = 10.

**Figure 12:**
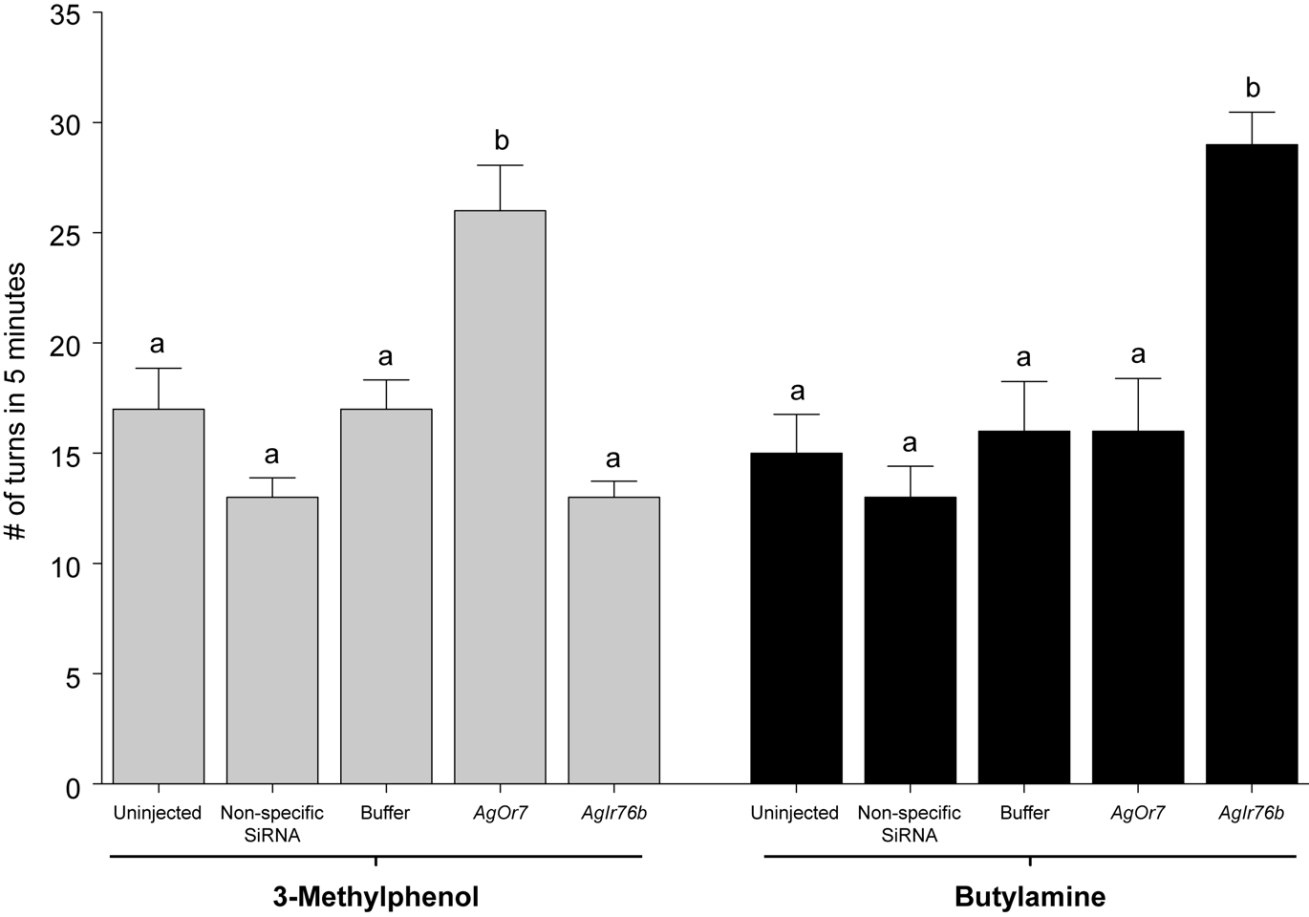
Olfactory responses to 3-methylphenol and butylamine are mediated by distinct signaling pathways. The turning rates exhibited by uninjected larvae as well as those receiving mock (buffer-alone), non-specific, or siRNA injections in response to 10^−4^ v/v dilutions of 3-methylphenol or butylamine were assessed independently over a 5 min time lapse. (A) Larval responses to 3-methylphenol were significantly altered by *AgOr7* knockdown but unaffected by *AgIr* silencing. (B) Conversely, responses to butylamine were sensitive to reduction in *AgIr76b* mRNA levels but indifferent to silencing of *AgOr7* expression.

**Figure 13:**
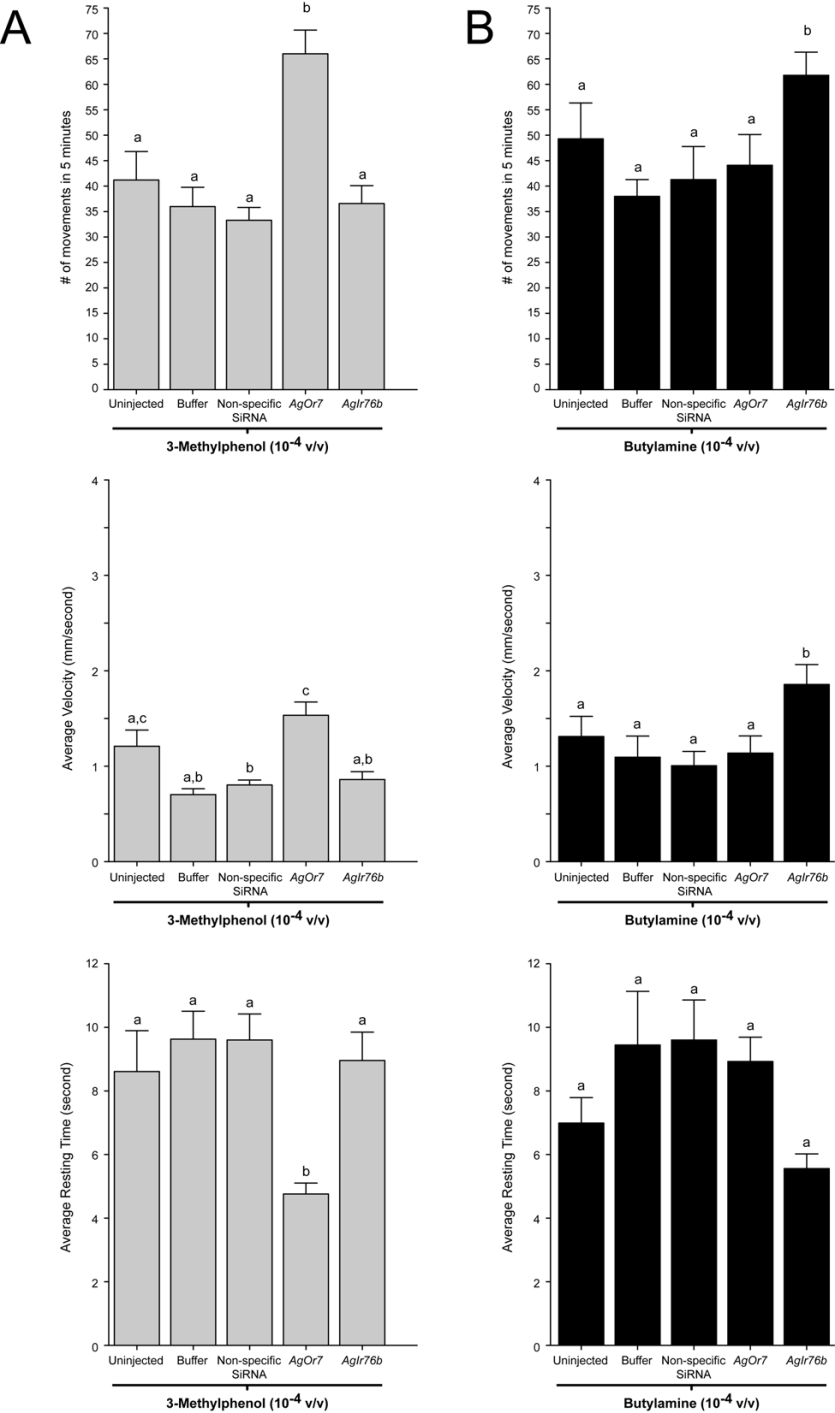
Odorant-specific differential effects of *AgOr*/*AgIr*knockdown. Averaged responses of buffer, non-specific, *AgOr7*, *and AgIr76b*-siRNA injected larvae in the presence of 10^−4^ v/v dilutions of 3-methylphenol (3MP, left panels) or butylamine (BA, right panels). Histograms of larval movement (A), velocity (B), and resting time (C) are presented. Knockdown *AgOr7* mRNA in larvae displayed significant behavioral alterations in response to 3MP without affecting BA-evoked behavior. Conversely, reduction of *AgIr76b* levels altered larval responses to BA without significantly affecting 3MP responses. Alteration of behavioral responses did not occur in the controls (*p*<0.05). Results are shown as means ± SE, *n* = 10.

In order to examine whether AgORs and AgIRs perform distinct functional roles in the olfactory system of *An. gambiae*, we carried out behavioral assays using two additional unitary odorants that have been used successfully in previous behavioral and functional studies [14]. The first was 3-methylphenol (3MP), which was shown to activate AgOR-dependent pathways and evoke robust behavioral responses in larvae [14]. In our current studies, larvae manifest dose-dependent reductions in turns and overall movement, as well as threshold-dependent increases in average resting time (Figure 11). Furthermore, larval responses to 10^−4^ dilutions of 3MP were significantly altered in larvae injected with *AgOr7* siRNA, whereas control or buffer-injected larval responses were statistically equivalent to uninjected control larvae (Figures 12 and 13A). AgOR40 is one of 3 larval AgORs with a demonstrated sensitivity to 3MP [14]. In that light, we also tested the ability of siRNA mediated silencing of *AgOr40* expression to alter larval responses to 3MP—in these studies a marginal but not statistically significant effect was observed (unpublished data) that is consistent with the role of multiple AgORs in mediating larval sensitivity to 3MP.

The next set of studies employed butylamine, a unitary odorant which has been shown to activate grooved-peg ORNs in *An. gambiae* [23] and *Culex quinquefasciatus* mosquitoes [24]. As was the case for 3MP, uninjected *An. gambiae* larvae displayed robust dose-dependent responses to butylamine (Figure 11). In contrast to the *AgOr7*-dependent nature of larval responses to 3MP, larval responses to butylamine were indistinguishable among animals treated with *AgOr7* and control siRNAs or microinjected with buffer alone (Figures 12 and 13B).

Based on their homology to DmIRs, which have been shown to mediate responses to amines and other odorants in Drosophila [12], we postulated that AgIRs mediate larval responses to butylamine. To test this hypothesis, siRNA-mediated gene knockdowns were used in an attempt to silence larval AgIRs and subsequently examine the responses of larvae to butylamine. Of the *AgIrs* tested, microinjection of only one—*AgIr76b*—displayed siRNA-specific effects on larval responses to butylamine. Microinjection of *AgIr76b* siRNAs reduced *AgIr76b* mRNA levels (Figure S2) and led to significant alterations in larval responses to butylamine (Figures 12 and 13B). Larval responses to butylamine were unaffected in *AgOr7* knockdowns and by microinjection of non-specific siRNAs or buffer-alone controls (Figures 12 and 13B).

## Discussion

In the face of a dearth of traditional genetic tools and a robust transgenic capacity, the ability to carry out RNAi-mediated gene silencing on individual *An. gambiae* larva provides an opportunity to examine the molecular basis for olfactory driven behaviors in this disease vector. Furthermore, the relative simplicity of the larval nervous system provides a considerably more tractable model within a non-model system for understanding similar processes that are presumed to underlie chemosensory responses in adults that directly contribute to anopheline vectorial capacity.

In this study, we have developed a simple behavioral paradigm that can be used to track the olfactory responses of individual *An. gambiae* larva to a range of chemical stimuli. Overall, these data are consistent with the hypothesis that when larvae are exposed to a repellent compound, such as DEET, they exhibit an increased rate of turning and a rise in overall movement and velocity. In contrast, an attractant such as yeast paste or 3MP leads to a reduction in the number of movements, turns, and average velocity while the average resting time is increased.

Together with gene-silencing approaches, we have employed a novel behavioral assay to provide compelling in vivo evidence that, for the first time, supports a direct in vivo role of AgORs in olfactory processes in *An. gambiae*. Furthermore, these studies go further to address the molecular mechanism responsible for DEET mediated repulsion of insects. Previous studies [25] suggesting that DEET's mode of action is to inhibit the activation of a subset of insect ORs that would otherwise be activated by attractants are in contrast to models that suggest DEET acts via direct excitation of OR-expressing ORNs that, in turn, evoke downstream behavioral repulsion. The excito-repellent hypothesis is consistent with our previous study on the larval olfactory system in *An. gambiae* [14] that showed robust DEET-mediated behavioral responses that correlated with a discrete population of larval ORNs co-expressing AgOR7/AgOR40 as well as specific DEET stimulation of Xenopus oocytes injected with AgOR7/AgOR40 cRNAs. This hypothesis is also supported by other studies that describe DEET-mediated activation of a subset of ORNs in *Culex* mosquitoes [26] and more recent work in *Aedes aeqypti* suggesting that DEET sensitivity is a genetically determined characteristic affecting the functionality of discrete ORNs [27]. While the reduction in DEET-mediated repellent responses in larvae undergoing RNAi mediated silencing of *AgOr7* is consistent with a general requirement for AgOR-based signaling, the similar effects of *AgOr40* silencing specifically supports the role of both these molecular targets in mediating DEET repellency. That these behavioral effects were manifest by DEET alone, i.e. in the absence of any other stimuli, further validates our earlier study and supports a direct excito-repellent mechanism for DEET activity.

Lastly, these studies uncover the existence of at least two parallel chemosensory transduction systems in larval-stage *An. gambiae* that respond to distinct classes of odorant stimuli. One pathway, which is in keeping with the established literature for insect olfactory signal transduction, is based on the obligatory role of the non-conventional anopheline Or83b family member *AgOr7*, which acts together with other conventional AgORs in the formation of functional receptors. It is likely that AgOR-dependent signaling pathways impact responses to a wide range of odorant cues that play important roles in several aspects of anopheline behavior. These pathways are exemplified by the dramatic alterations in the DEET and 3MP responses of *An. gambiae* larvae after RNAi-mediated silencing of *AgOr7* transcripts (Figures 5, 12). The other pathway depends on the function of the *AgIr* gene family, which likely recognizes different odor classes than the *AgOr* pathway. Moreover, the similarities between AgIRs8a and 25a and iGluRs suggest that cellular receptors for glutamate in the antenna could act as a neuromodulator of ORN function. This hypothesis is consistent with the inability of *AgIr25a* siRNAs to alter larval behavioral responses to odors (unpublished data).

Recent functional analyses [17],[18] of AgOR-based odor coding against a diverse panel of compounds suggest that, in *An. gambiae*, olfactory pathways respond to a wide range of odorant stimuli with particular affinity for heterocyclics and aromatics that are associated with human skin emanations [15],[16]. These groups of odorants are thought to play essential roles in host-seeking, oviposition, and other behaviors that are critical for anopheline life cycles [28]. Coincidently, this AgOR-based odor space is characterized by sparse responses to the majority of acids, aldehydes, and esters that were tested in addition to being particularly devoid of amine-elicited responses [17],[18]. This raised the suggestion that sensitivity to these classes of odorants might lie outside of *AgOr*-dependent olfactory signaling pathways.

We have identified several *AgIrs* that are expressed in larval olfactory tissues (Figure 10) and have used RNAi-mediated gene silencing to demonstrate the role of one of these genes in mediating larval responses to the AgOR-independent odorant butylamine. Critically, while knockdown of *AgIr76b* specifically altered larval responses to butylamine, there was no effect on responses to two other unitary odorants that were dependent on *AgOr7* expression. These data are consistent with the hypothesis that, in contrast to the AgOR-dependent sensitivity to 3MP, DEET, and a broad range of “general” odorants [17],[18], anopheline responses to other odorants (e.g., butylamine) are mediated through *AgIr*-dependent signaling. There is reason to assume that these parallel pathways persist through to adult *An. gambiae* where *AgIrs* are likely to be responsible for olfactory sensitivity to important human kairomones, such as ammonia and lactic acid that are known to activate ORNs in grooved peg sensilla [29] that are devoid of AgOR7 [5]. Indeed, we have observed expression of multiple *AgIrs* in adult olfactory appendages, supporting the hypothesis that this family of genes is involved in chemosensory signaling in adults (manuscript in preparation).

Current efforts are directed toward expanding our understanding of *AgIr*-based odor coding in *An. gambiae*. Improving our understanding of olfactory signal transduction in *An. gambiae* may lead to new opportunities to target olfactory mediated behaviors at the molecular level. In turn, this may reduce the vectorial capacity of *An. gambiae* and help reduce the transmission of malaria and other important human diseases.

## Supporting Information

Figure S1 **Quantitative analysis demonstrates significant transcript level reduction of** ***AgOr7*** **and** ***AgOr40*** **after siRNA treatment.** Larval cDNAs for qRT-PCR were generated using equal amounts (2 µg for AgOr7 and 4 µg for AgOr40) of RNA extracted from hand-dissected larval heads from each injection treatment group, and three technical replicates were performed for each experimental group. *AgOr7* and *AgOr40* mRNA levels were quantified as fold-changes relative to *Rps7* using the method of Pfaffl [20]. *AgOr7* and *AgOr40* levels are shown after normalization to buffer-alone controls in each of three experimental replicates. Histograms showing averaged *AgOr7* and *AgOr40* levels normalized to buffer-alone injection controls. Standard errors were ±0.041 and ±0.029 for non-specific and *AgOr7* siRNA injections; ±0.127 and ±0.392 for non-specific and *AgOr40* siRNA injections, respectively. Raw data from each qRT-PCR reaction indicating cycle-threshold (CT) and primer efficiency information for each technical replicate.
(0.71 MB JPG)

Figure S2 **Quantitative mRNA analysis demonstrates significant transcript level reduction of** ***AgIr76b*** **after siRNA treatment.** Larval cDNAs for qRT-PCR were generated using equal amounts (∼3.5 µg) of RNA extracted from hand-dissected larval heads from each injection treatment group. Two independent biological replicates were performed, each consisting of three technical replicates for every experimental group. *AgIr76b* mRNA levels were quantified as fold-changes relative to *Rps7* using the method of Pfaffl [20]. *AgIr76b* levels are shown as averaged values of both biological replicates after normalization to buffer alone controls in each of three technical replicates. Histograms showing averaged *AgIr76b* levels normalized to buffer alone injection controls. Standard errors were ±0.04 and ±0.003 for non-specific and *AgIr76b* siRNA injections, respectively. Raw data from each qRT-PCR reaction indicating cycle-threshold (CT) and primer efficiency information for each biological/technical replicate.
(0.38 MB JPG)

Table S1 **Annotation of AgIR family members.** Nomenclature, chromosome positions, and conceptual peptide sequences of ionotropic glutamate (AgamGLUR and AgamNMDAR) and variant ionotropic glutamate receptor (AgamGLUvir) families in *An. gambiae*. Column headers indicate: (1) long form of gene name; (2) short form of peptide name; (3) VectorBase gene identification number; (4) chromosome location and base pair position (plus, + or minus, − strand in parentheses) of updated gene annotation; and (5) conceptual peptide sequence of new gene model (single letter amino acid code). AgGLURI and AgGLURIIb represent partial peptides where the 5′ end of the gene has not been annotated.
(0.08 MB XLS)

## Abbreviations

3MP: 3-methyl-phenol
AgIRs: *An. gambiae* ionotropic glutamate receptors
AgORs: *An. gambiae* odorant receptors
DEET: N, N-diethyl-m-toluamide
ORN: olfactory receptor neuron
RNAi: RNA interference
siRNA: small interfering RNA
