# Supplementary figures and images for: Distinct Olfactory Signaling Mechanisms in the Malaria Vector Mosquito Anopheles gambiae
Source: PLoS Biol. 2010 Aug 31;8(8):e1000467. doi: 10.1371/journal.pbio.1000467 (PMC2930861; doi:10.1371/journal.pbio.1000467)

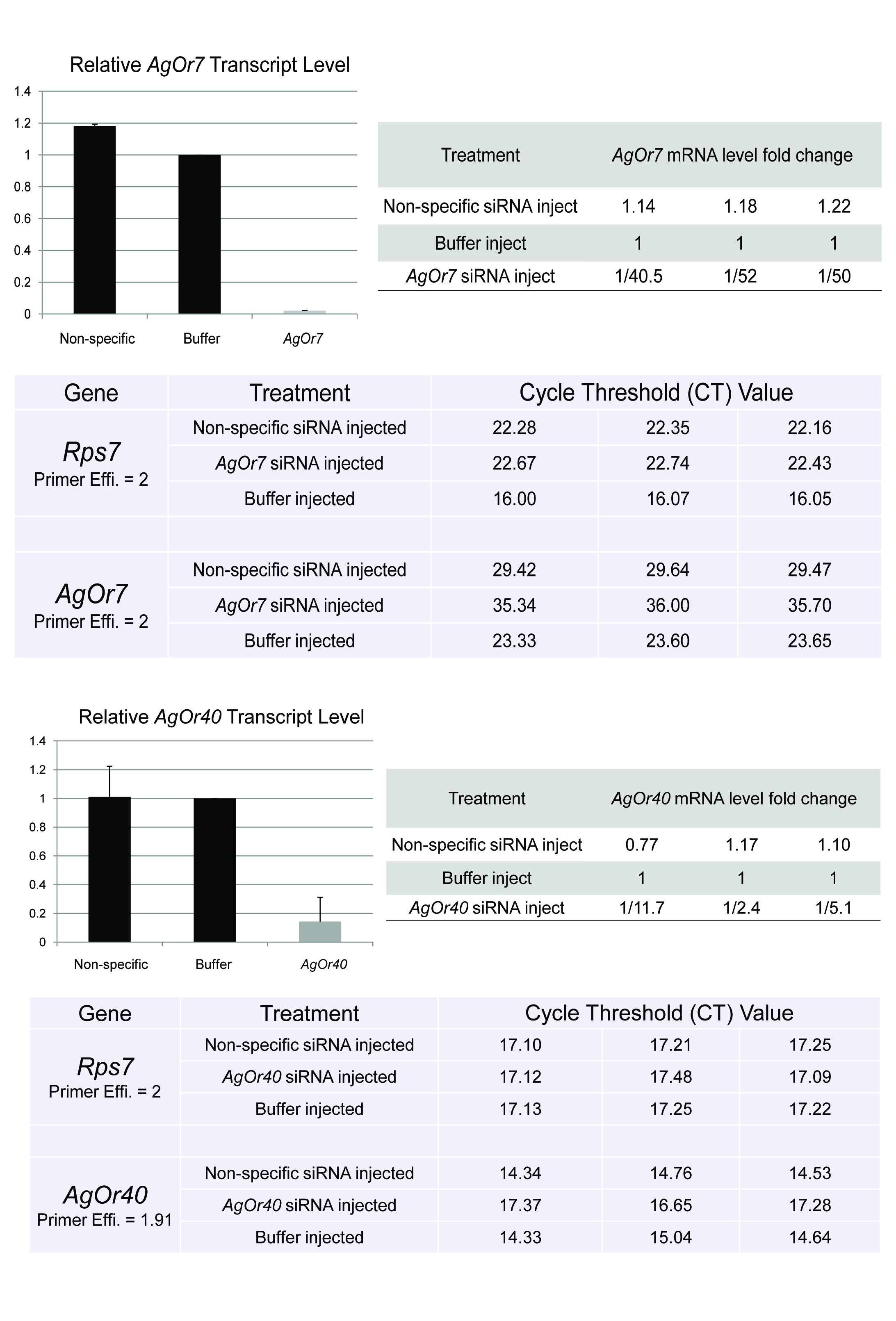

Supplement: Figure S1 — Quantitative analysis demonstrates significant transcript level reduction of AgOr7 and AgOr40 after siRNA treatment. Larval cDNAs for qRT-PCR were generated using equal amounts (2 µg for AgOr7 and 4 µg for AgOr40) of RNA extracted from hand-dissected larval heads from each injection treatment group, and three technical replicates were performed for each experimental group. AgOr7 and AgOr40 mRNA levels were quantified as fold-changes relative to Rps7 using the method of Pfaffl [20]. AgOr7 and AgOr40 levels are shown after normalization to buffer-alone controls in each of three experimental replicates. Histograms showing averaged AgOr7 and AgOr40 levels normalized to buffer-alone injection controls. Standard errors were ±0.041 and ±0.029 for non-specific and AgOr7 siRNA injections; ±0.127 and ±0.392 for non-specific and AgOr40 siRNA injections, respectively. Raw data from each qRT-PCR reaction indicating cycle-threshold (CT) and primer efficiency information for each technical replicate. (0.71 MB JPG) [file pbio.1000467.s001.jpg]

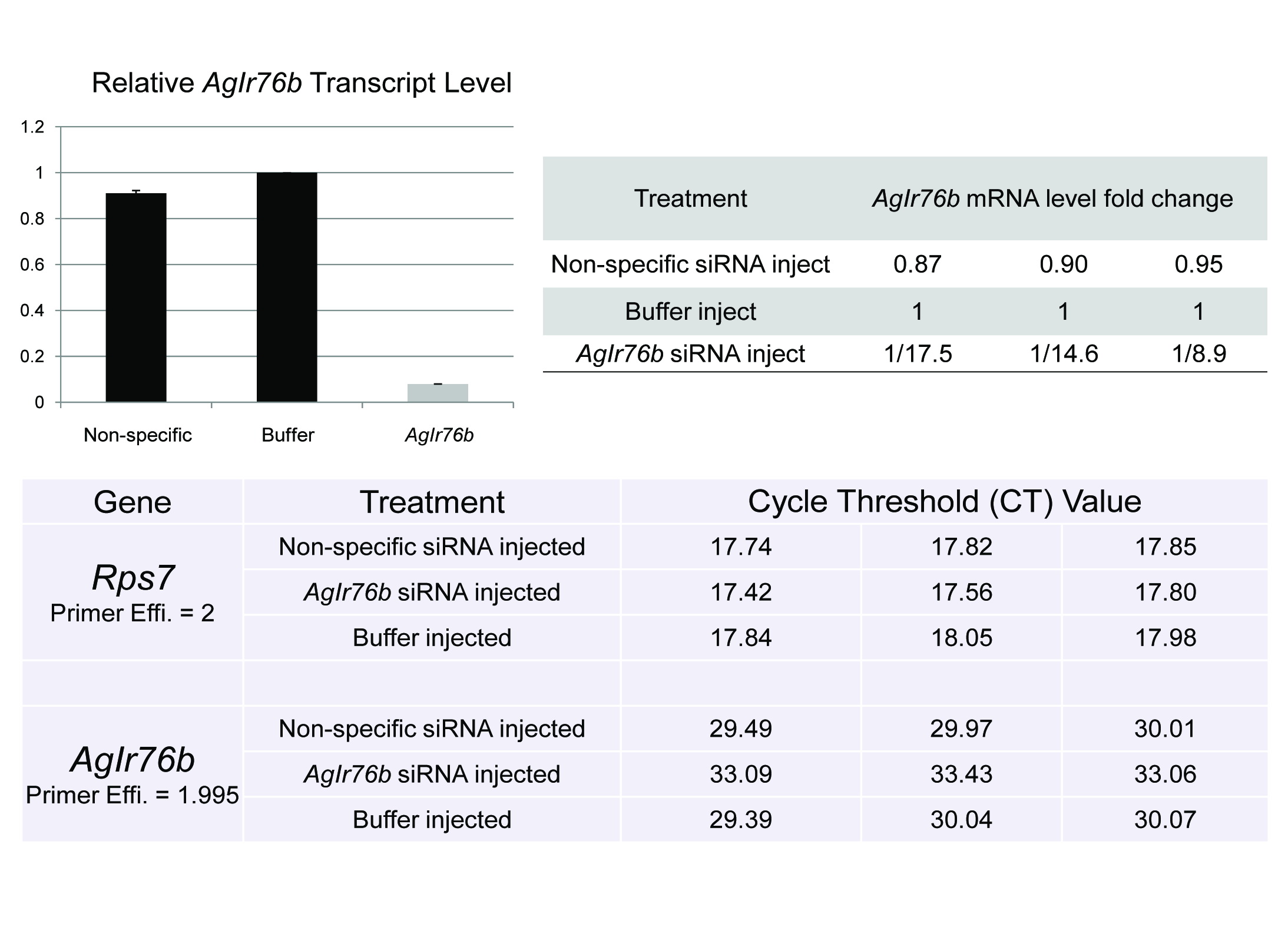

Supplement: Figure S2 — Quantitative mRNA analysis demonstrates significant transcript level reduction of AgIr76b after siRNA treatment. Larval cDNAs for qRT-PCR were generated using equal amounts (∼3.5 µg) of RNA extracted from hand-dissected larval heads from each injection treatment group. Two independent biological replicates were performed, each consisting of three technical replicates for every experimental group. AgIr76b mRNA levels were quantified as fold-changes relative to Rps7 using the method of Pfaffl [20]. AgIr76b levels are shown as averaged values of both biological replicates after normalization to buffer alone controls in each of three technical replicates. Histograms showing averaged AgIr76b levels normalized to buffer alone injection controls. Standard errors were ±0.04 and ±0.003 for non-specific and AgIr76b siRNA injections, respectively. Raw data from each qRT-PCR reaction indicating cycle-threshold (CT) and primer efficiency information for each biological/technical replicate. (0.38 MB JPG) [file pbio.1000467.s002.jpg]
